# Risk factors associated with the development of postpartum diabetes in Japanese women with gestational diabetes

**DOI:** 10.1186/s12884-017-1654-4

**Published:** 2018-01-08

**Authors:** Yukari Kugishima, Ichiro Yasuhi, Hiroshi Yamashita, So Sugimi, Yasushi Umezaki, Sachie Suga, Masashi Fukuda, Nobuko Kusuda

**Affiliations:** Department of Obstetrics and Gynecology, NHO Nagasaki Medical Center, 1001-1 2-chome Kubara, Omura City, Nagasaki 856-8562 Japan

**Keywords:** Diagnostic criteria, Gestational diabetes, HbA1c, Predictive factors, Postpartum diabetes

## Abstract

**Background:**

Although the onset of gestational diabetes (GDM) is known to be a significant risk factor for the future development of type 2 diabetes, this risk specifically in women with GDM diagnosed by the International Association of Diabetes and Pregnancy Study Group (IADPSG) criteria has not yet been thoroughly investigated. This study was performed to investigate the risk factors associated with the development of postpartum diabetes in Japanese women with a history of GDM, and the effects of the differences in the previous Japanese criteria and the IADPSG criteria.

**Methods:**

This retrospective cohort study included Japanese women with GDM who underwent at least one postpartum oral glucose tolerance test (OGTT) between 2003 and 2014. Cases with overt diabetes in pregnancy were excluded. We investigated the risk factors including maternal baseline and pregnancy characteristics associated with the development of postpartum diabetes.

**Results:**

Among 354 women diagnosed with GDM during the study period, 306 (86%) (116/136 [85.3%] and 190/218 [87.2%] under the previous criteria and the IADPSG criteria, respectively) who underwent at least 1 follow-up OGTT were included in the study. Thirty-two women (10.1%) developed diabetes within a median follow-up period of 57 weeks (range, 6–292 weeks). Eleven (9.5%) and 21 (11.1%) were diagnosed as GDM during pregnancy based on the previous Japanese criteria and the IADPSG criteria, respectively, which did not significantly differ between those criteria. A multivariate logistic regression analysis revealed that HbA1c and 2-h plasma glucose (PG) at the time of the diagnostic OGTT during pregnancy were independent predictors of the development of diabetes after adjusting for confounders. The adjusted relative risk of HbA1c ≥5.6% for the development of diabetes was 4.67 (95% confidence interval, 1.53-16.73), while that of 2-h PG ≥183 mg/dl was 7.02 (2.51-20.72).

**Conclusions:**

A modest elevation of the HbA1c and 2-h PG values at the time of the diagnosis of GDM during pregnancy are independent predictors of the development of diabetes during the postpartum period in Japanese women with a history of GDM. The diagnostic criteria did not affect the incidence of postpartum diabetes.

## Background

The onset of gestational diabetes (GDM) during pregnancy is known to be a significant risk factor for the future development of type 2 diabetes. The odds ratio, in comparison to patients who are normoglycemic during pregnancy, is 7.43 [1]. Although evidence to support this was already published in 1978 [2], it has received much more attention in the background of the recent worldwide pandemic of diabetes and obesity [3]. In this background, women with a history of GDM have been becoming a key target population in efforts to prevent the future development of diabetes.

In 2010, new international diagnostic criteria for GDM were published [4]. The criteria were based on evidence of the perinatal outcomes. Thus, it has not been well investigated whether women with GDM who are diagnosed according to the IADPSG criteria are at a similarly high risk of developing postpartum diabetes. We previously reported that there was no significant difference in the effect of the early postpartum development of impaired glucose tolerance between the IADPSG criteria and the previous Japanese criteria [5]. However, it is still not clear whether the differences in the diagnostic criteria affect the risk of the future development of diabetes.

The introduction of the IADPSG criteria has resulted in a 3-fold increase in the prevalence of GDM in the Japanese population [6]. Thus, a more efficient follow-up system is necessary for screening for postpartum diabetes. The triage of high-risk women to more intensive follow-up protocols seems to be more relevant. There seem to be a large number of risk factors, including maternal characteristics and pregnancy factors that are linked to the future development of type 2 diabetes in women with a history of GDM [7]. The maternal characteristics include maternal obesity, a family history of diabetes, ethnicity, advanced maternal age. The pregnancy factors include, but are not limited to, an early diagnosis of GDM, fasting hyperglycemia, an elevated HbA1c value, and insulin use [7]. In our previous study, we aimed to demonstrate the risk factors associated with abnormal glucose tolerance at 6–8 weeks postpartum [5]. However, thus far, no studies of Japanese subjects that followed up patients beyond the early postpartum period have been reported.

In this current study, we aimed to identify the risk factors associated with the development of diabetes in Japanese women with a history of GDM over a longer postpartum period, and to investigate whether the differences between the previous Japanese criteria and the IADPSG criteria influence the risk of the development of postpartum diabetes.

## Methods

In this retrospective cohort study of a single perinatal care center in Japan, we obtained data for women with GDM who underwent postpartum 75-g oral glucose tolerance tests (OGTT) at the National Hospital Organization Nagasaki Medical Center (Omura, Japan) between January, 2003 and December, 2014. We used two different diagnostic criteria during this period: the Japan Society of Obstetrics and Gynecology (JSOG) criteria [8], which were used until June 2010; and the IADPSG criteria, which were used from July 2010 (Table 1). Because of the possibility of pregestational diabetes, we excluded women who were diagnosed with overt diabetes during pregnancy according to the IADPSG criteria [4], including those with a fasting plasma glucose level of ≥126 mg/dl or an HbA1c level of ≥6.5% on an OGTT during pregnancy. We only included women of Japanese ethnicity in the present study. In both of the study periods with different diagnostic criteria, to screen for GDM during pregnancy, we performed universal screening of all pregnant women using a 50-g glucose challenge test around 24 weeks’ gestation; those with values of ≥135 mg/dL underwent a diagnostic 75-g oral glucose tolerance test (OGTT) after overnight fasting. We also measured the HbA1c values at the time of the diagnostic OGTT.

**Table 1:** The diagnostic criteria using the 75-g 2-h OGTT in Japan

	GDM		Postpartum diabetes
Diagnostic criteria	JSOG criteria [2]	IADPSG criteria [1]	WHO criteria [3]
Glucose load	75 g	75 g	75 g
Time of the diagnosis	Until June 2010	From June 2010	
Fasting PG	≥100 mg/dl	≥92 mg/dl	≥126
1-h PG	≥180 mg/dl	≥180 mg/dl	N/A
2-h PG	≥150 mg/dl	≥153 mg/dl	≥200
Required to diagnose GDM	Two or more abnormal values	One abnormal value or more	One abnormal value or more

*OGTT* oral glucose tolerance test, *GDM* gestational diabetes, *JSOG* Japan Society of Obstetrics and Gynecology, *IADPSG* Internal Association of Diabetes and Pregnancy Study Group, *WHO* World Health Organization, *PG* plasma glucose, *N/A*, not addressed

We used the standard treatment practices for women with GDM, including diet and insulin therapy based on the results of the self-monitored blood glucose (SMBG) level. Insulin therapy was prescribed if the patient achieved less than 80% of the target blood glucose levels, including fasting and 2-h postprandial blood glucose levels of <95 mg/dl and <120 mg/dl, respectively. We did not prescribe any oral hypoglycemic agents during pregnancy or the postpartum period.

Women with a history of GDM underwent the first follow-up OGTT at 6–8 weeks postpartum; thereafter, the test was then repeated every 6–12 months. We defined postpartum diabetes according to the WHO criteria [9] (Table 1).

We obtained the patients’ basic maternal characteristics including their age, pre-pregnancy body mass index (BMI), and family history of diabetes (defined as unspecified diabetes among first- and second-degree relatives). We also obtained data related to their pregnancy, including the gestational age (GA) at the time of the diagnostic OGTT, the plasma glucose (PG) and HbA1c levels at the time of the diagnostic OGTT, the requirement of insulin therapy, and weight gain throughout pregnancy.

The primary outcome measure was postpartum development of diabetes. We investigated the association between the primary outcome measure and the risk factors, including the basic maternal and perinatal characteristics. We first used a univariate logistic regression analysis to test the association between each risk factor and the postpartum development of diabetes. Factors with a *p* value of <0.05 on the univariate analysis were included in a multivariate logistic regression analysis. The multivariate logistic regression analysis was used to test for independent associations between risk factors and the development of diabetes. In the multivariate analysis, we converted the factors that were numerically associated with the development of postpartum diabetes into categorical variables as a clinical viewpoint. A receiver operator characteristic (ROC) curve was used to identify the optimum cut-off values for those variables. We also used Student’s *t*-test and a chi-squared test to compare numerical variables and the difference in ratios between groups, respectively. *P* values of <0.05 were considered to indicate statistical significance. This study was conducted with the approval of the Institution Review Board of Nagasaki Medical Center to collect the clinical data with informed consent.

## Results

We included 306 women who underwent at least one postpartum follow-up OGTT. In the same period, 354 women were diagnosed with GDM, including 136 and 218 by the JSOG and the IADPSG criteria, respectively. Among the patients who underwent at least 1 postpartum OGTT, 116 (38%) and 190 (62%) women were diagnosed according to the JSOG and IADPSG criteria, respectively. Thus, the follow-up rate (defined by the performance of at least 1 postpartum OGTT) was 86% (306/354) in total subjects and 85.3% (116/136) and 87.2% (190/218) in the JSOG and the IADPSG criteria, respectively. The maternal characteristics of the patients in each group and the results of their diagnostic OGTTs during pregnancy are shown in Table 2. The PG levels during the diagnostic OGTT were significantly higher in women during the JSOG period (JSOG group) than they were during the IADPSG period (IADPSG group). The rates of women who underwent two or more follow-up OGTTs in the JSOG and IADPSG periods were 70% and 78%, respectively, and did not differ to a statistically significant extent. More than half of the women underwent an OGTT at more than one year postpartum. There was a significant difference in the length of the follow-up period between the two groups (Table 2).

**Table 2:** The maternal characteristics and 75-g OGTT results during pregnancy in terms of the different diagnostic criteria

Variables	All subjects (*n* = 306)	Postpartum OGTT		
		JSOG criteria (*n* = 116)	IADPSG Criteria (*n* = 190)	*P* value *
Maternal age (years)	33.0 ± 5.1	33.2 ± 4.8	32.9 ± 5.2	0.52
Nulliparous (%)	136 (44%)	46 (40%)	90 (47%)	0.19
Family history of diabetes (%)	124 (41%)	47 (41%)	77 (41%)	1.0
Pre-pregnancy BMI (kg/m^2^)	23.5 ± 4.8	24.0 ± 4.9	23.2 ± 4.8	0.14
Pre-pregnancy BMI ≥25 kg/m^2^	92 (30%)	41 (35%)	52 (27%)	0.14
GA at OGTT (weeks)	24.2 ± 6.7	23.9 ± 7.6	24.4 ± 6.1	0.60
OGTT results during pregnancy				
Fasting PG (mg/dl)	86 ± 10	88 ± 11	85 ± 10	0.0046
1-h PG (mg/dl)	186 ± 27	197 ± 23	179 ± 26	<0.0001
2-h PG (mg/dl)	161 ± 26	168 ± 22	156 ± 27	0.0001
HbA1c (%)	5.5 ± 0.4 (*n* = 269)	5.5 ± 0.4 (*n* = 108)	5.5 ± 0.4 (*n* = 158)	0.94
Insulin therapy during pregnancy (%)	162 (53%)	54 (47%)	108 (58%)	0.057
Weight gain during pregnancy (kg)	7.3 ± 5.1	6.9 ± 5.8	7.5 ± 4.6	0.37
Mean follow-up period (weeks) (median, range)	68 ± 61 (57, 7–292)	83 ± 81 (58, 7–292)	59 ± 43 (57, 7–164)	0.0006
At least two follow-up OGTTs (%)	229 (75%)	81 (70%)	148 (78%)	0.079
More than 12 months of follow-up OGTTs (%)	165 (54%)	61 (53%)	104 (55%)	0.19
Women who developed diabetes (%)	32 (10.5%)	11 (9.5%)	21 (11.1%)	0.66

* P values represent comparisons between the JSOG and IADPSG criteria using Student’s *t*-test or a chi-squared test

*OGTT* oral glucose tolerance test, *JSOG* Japan Society of Obstetrics and Gynecology, *IADPSG* Internal Association of Diabetes and Pregnancy Study Group, *BMI* body mass index, *GA* gestational age, *PG* plasma glucose

During the mean follow-up period of 68 ± 61 weeks (median, 57 weeks; range, 7–292 weeks), 32 (10.5%) women developed diabetes within a follow-up period of 59 ± 53 weeks (median, 47 weeks; range, 7–230 weeks). This rate was not significantly different from that of the women who did not develop diabetes (mean, 69 ± 62 weeks; median, 58 weeks; range 7–292 weeks). Eleven (9.5%) and 21 (11.1%) women with diabetes were included in the JSOG and IADPSG groups, respectively (Table 2); the incidence was not different between the different diagnostic criteria group even after adjusting for the follow-up period. Regarding the time period from the index delivery to the onset of diabetes in those who developed diabetes, women diagnosed under the IADPSG criteria developed diabetes significantly sooner than those diagnosed under the JSOG criteria (44 ± 26 vs. 88 ± 78 weeks, *p* = 0.024).

The women who developed postpartum diabetes were more obese before pregnancy (*p* = 0.0032), showed elevated 2-h PG (*p* = 0.016) and HbA1c (*p* < 0.0001) levels at the time of the diagnostic OGTT, and required more insulin therapy during pregnancy (*p* = 0.0031) in comparison to those who did not develop diabetes during the study period (Table 3). A univariate logistic regression analysis revealed that a higher pre-pregnancy BMI (*p* = 0.0044), 2-h PG (*p* = 0.016), HbA1c (*p* < 0.0001), and the requirement of insulin therapy (*p* = 0.0031) were significant risk factors for the postpartum development of diabetes (Table 4). In multivariate regression models that used the variables that were identified as significant in the univariate analysis, we found that only the 2-h PG and HbA1c levels were independent predictors of the development of diabetes during the postpartum period (Table 5). The association remained significant after controlling for maternal age, parity, a family history of diabetes, the GA and fasting and 1-h PG at the OGTT, weight gain during pregnancy, and the follow-up period (Table 5). Because fasting and the 1-h PG showed near-significance in the univariate analysis (Table 4), we also examined the association between those two variables and the development of diabetes by a multivariate analysis including these two variables in addition to the four significant variables and found that neither fasting nor the 1-h PG was significantly associated with the postpartum disorder.

**Table 3:** The maternal characteristics and 75-g OGTT results during pregnancy: The difference between women who developed postpartum diabetes and those who did not

	Diabetes (*n* = 32)	Non-diabetes (*n* = 274)	*P* value^*^
Maternal age (years)	34.3 ± 4.6	32.9 ± 5.1	0.14
Nulliparous (%)	11 (34%)	125 (46%)	0.22
Family history of diabetes (%)	13 (41%)	110 (41%)	1.0
Pre-pregnancy BMI (kg/m^2^)	25.9 ± 5.7	23.2 ± 4.7	0.0032
Pre-pregnancy BMI ≥25 kg/m^2^	17 (53%)	76 (28%)	0.0031
GA at OGTT (weeks)	23.8 ± 7.9	24.3 ± 6.6	0.71
JSOG criteria period	11 (34%)	105 (38%)	0.66
OGTT results during pregnancy			
Fasting PG (mg/dl)	89 ± 11	86 ± 10	0.091
1-h PG (mg/dl)	195 ± 25	185 ± 27	0.056
2-h PG (mg/dl)	172 ± 32	160 ± 25	0.016
HbA1c (%) (*n* = 269)	5.8 ± 0.4 (*n* = 29)	5.5 ± 0.4 (*n* = 240)	<0.001
Insulin therapy during pregnancy (%)	25 (78%)	137 (51%)	0.0031
Weight gain during pregnancy (kg)	7.6 ± 3.9	7.2 ± 5.2	0.72
Follow-up period (weeks) (median, range)	59 ± 53 (47, 7–230)	69 ± 62 (58, 7–291)	0.39
At least two follow-up OGTT (%)	26 (81%)	203 (74%)	0.63
More than 12 months follow-up OGTT (%)	14 (44%)	151 (55%)	0.40

* P values represent comparisons between women who developed diabetes and those who did not using Student’s *t*-test or a chi-squared test

*BMI* body mass index, *GA* gestational age, *OGTT* oral glucose tolerance test, *JSOG* Japan Society of Obstetrics and Gynecology, *PG* plasma glucose

**Table 4:** The association between the predictive variables and the postpartum development of diabetes in a univariate logistic regression analysis

Predictive Variables	Chi-square	*P* value
Maternal age (years)	2.15	0.14
Nulliparous (%)	1.47	0.22
Family history of diabetes (%)	0.0	0.99
Pre-pregnancy BMI (kg/m^2^)	8.13	0.0044
Pre-pregnancy BMI ≥25 kg/m^2^	8.77	0.0031
GA at OGTT (weeks)	0.13	0.71
JSOG criteria period^a^	0.19	0.66
OGTT results during pregnancy		
Fasting PG (mg/dl)	2.82	0.093
1-h PG (mg/dl)	3.66	0.056
2-h PG (mg/dl)	5.76	0.016
HbA1c (%) (n = 269)	16.3	<0.0001
Insulin therapy in pregnancy (%)	8.75	0.0031
Weight gain during pregnancy (kg)	0.13	0.72
Follow-up period (weeks)	0.74	0.39

*BMI* body mass index, *GA* gestational age, *OGTT* oral glucose tolerance test, *JSOG*, Japan Society of Obstetrics and Gynecology, *PG* plasma glucose

^a^adjusted for the follow-up period

**Table 5:** Results of the multiple logistic regression analysis to investigate the factors associated with the postpartum development of diabetes: The continuous variable model (*n* = 269)^a^

Variables included in the multivariate models	Model 1			Model 2^b^		
	RR	95% CI	*P* value	RR	95% CI	*P* value
Pre-pregnancy BMI (kg/m^2^)	1.04	0.96–1.13	0.29	1.08	0.98–1.20	0.13
2-h PG (mg/dl)	1.02	1.00–1.03	0.042	1.02	1.00–1.04	0.030
HbA1c (%)	5.38	1.64–19.06	0.0069	5.04	1.27–22.0	0.021
Insulin therapy during pregnancy (%)	1.92	0.71–5.78	0.22	1.92	0.66–6.46	0.24

^a^ We used data from 269 women who had HbA1c test results available at the time of the diagnostic OGTT during pregnancy

^b^ Adjusted for the maternal age, parity, family history of diabetes, GA at OGTT, fasting and 1-h PG, weight gain during pregnancy, and follow-up period

*RR* relative risk, *BMI* body mass index, *PG* plasma glucose, *OGTT* oral glucose tolerance test, *GA* gestational age

From the clinical point of view, we converted these numerical variables to categorical variables (Table 6). We used cutoff values of 183 mg/dl (area under the curve [AUC] 0.64) and 5.6% (AUC 0.74) for 2-h PG and HbA1c, respectively, which were derived from the ROC. A 2-h PG value of ≥183 mg/dl and an HbA1c value of ≥5.6% were significantly associated with the development of postpartum diabetes with an adjusted relative risk (RR) of 7.02 (95% confidence interval [CI] 2.51–20.72, *p* = 0.0002) and 4.67 (95% CI 1.53–16.73, *p* = 0.0061), respectively (Table 6).

**Table 6:** Multiple logistic regression models to investigate the association between the risk factors and the postpartum development of DM: The categorical variable model (*n* = 269)^a^

Variables included in the multivariate models	Model 1			Model 2 ^b^		
	RR	95% CI	P value	RR	95% CI	P value
Pre-pregnancy BMI ≥25 (kg/m^2^)	1.62	0.67–3.91	0.28	2.31	0.84–6.56	0.11
2-h PG ≥183 (mg/dl)	5.29	2.15–13.27	0.0004	7.02	2.51–20.72	0.0002
HbA1c ≥5.6 (%)	6.18	2.22–20.47	0.0003	4.67	1.53–16.73	0.0061
Insulin therapy during pregnancy (%)	2.02	0.75–6.10	0.17	2.30	0.75–8.17	0.15

^a^ We used data from 269 women who had HbA1c test results available at the time of the diagnostic OGTT during pregnancy

^b^ Adjusted for the maternal age, parity, family history of diabetes, GA at OGTT, fasting and 1-h PG, weight gain during pregnancy, and follow-up period

*RR* relative risk, *BMI* body mass index, *PG* plasma glucose, *OGTT* oral glucose tolerance test, *GA* gestational age

## Discussion

In this retrospective Japanese cohort study, 10.5% of women with a history of GDM developed diabetes during a median follow-up period of 57 weeks within up to 5 years. We also found that the 2-h PG and HbA1c values during the diagnostic 75-g OGTT in pregnancy were significant independent predictors of the postpartum development of diabetes, with an RR of 7.02 and 4.67, respectively, if a 2-h PG level of ≥183 mg/dl and an HbA1c value of ≥5.6% were used as cutoff values, after adjusting for the considerable confounders. With regard to the diagnostic criteria, there was no significant difference in the prevalence of the development of postpartum diabetes between the women who were diagnosed by the JSOG criteria (9.5%) and those who were diagnosed by the IADPSG criteria (11.1%).

To the best of our knowledge, this is the first report regarding the development of diabetes at more than one year postpartum in Japanese women with a history of GDM. In addition, among many follow-up studies of GDM patients, few studies have reported the prevalence of postpartum diabetes in women with a history of GDM who were diagnosed according to the IADPSG criteria. We found that the prevalence of postpartum diabetes in the IADPSG group was not significantly different to that in the JSOG group, although the mean follow-up period was significantly longer in the JSOG group (Table 2). In addition, in women diagnosed with postpartum diabetes, the duration from the index delivery to the development of diabetes was significantly shorter in the IADPSG group than in the JSOG group. Thus, in comparison to the previous criteria, the IADPSG criteria seemed to recognize more women who develop postpartum diabetes earlier. Assaf-Balut et al. [10] reported that the change in diagnostic criteria from the Carpenters-Coustan (CC) criteria to the IADPSG criteria did not affect the percentage of women with postpartum glucose disorder (29.5% vs. 32.3%, respectively [10]. Because the number of women who were diagnosed in the IADPSG group was higher than that in the CC group, the IADPSG criteria could be superior for identifying women with postpartum glucose disorder who would have been missed by the CC criteria [10], even though the IADPSG criteria are based on only the perinatal outcomes and not on the risk of developing postpartum diabetes.

There is already evidence to show that women with a history of GDM are at significant risk for the development of type 2 diabetes; however, the follow-up tests after delivery have been suboptimal [11, 12], in spite of the current recommendations including early postpartum diabetic screening at 6–12 weeks postpartum and further follow-up tests [13–15]. In addition to the markedly low follow-up rates of only 16–48% that were reported in previous studies [16–19], the increase in the number of women with GDM after the adoption of the IADPSG criteria makes their postpartum follow-up screening more difficult. Under these conditions, it is very important to identify women with a high risk of developing postpartum diabetes.

A recent meta-analysis using a univariate model identified a large number of risk factors for the future progression of diabetes. These included BMI, a family history of diabetes, non-white ethnicity, advanced maternal age, an early diagnosis of GDM, the fasting and post-glucose load PG, the HbA1c level, the use of insulin, multiparity, hypertensive disorder, and preterm delivery [7]. Although we did not investigate obstetric complications, such as hypertension and preterm delivery, or the breastfeeding conditions in our study, the risk factors identified in the univariate analysis were similar to those reported in Rayanagoudar’s study [7]. There were some differences between the studies regarding maternal age, family history, multiparity and GA at the time of their diagnosis. These differences are probably due to the small sample size of our study.

After controlling for confounders in the multivariate models, we identified two independent risk factors for the development of diabetes during a relatively long-term postpartum follow-up period of five years that were present during the index pregnancy: elevated 2-h PG and HbA1c values. Several authors have reported that the HbA1c level at the diagnosis of GDM during pregnancy is an independent predictor of the development of postpartum diabetes [20–22]. In a Swedish study [20] of 144 women with GDM who had high risk factors, including a first-degree family history of diabetes or previous GDM, the HbA1c and fasting PG values during pregnancy were found to be independent predictors in 43 cases (30.6%) in which women developed diabetes within 5 years postpartum. They found that an HbA1c level of ≥5.7% and a fasting PG level of ≥94 mg/dl (5.2 mmol/L) were associated with a 4.8- and 6.8-fold increase in the risk of developing postpartum diabetes, respectively, in comparison to women whose HbA1c and fasting PG levels were below these cutoff values. In our study, the cutoff HbA1c value derived from the ROC was 5.6%, which is in line with that in the Swedish study. We did not find a significant association between fasting PG and diabetes; instead, 2-h PG was an independent predictor. This is probably due to several factors, including the difference in the study populations, especially the fact that the Swedish study only included high-risk women, the difference in the lengths of the follow-up periods and ethnicity [23]. Despite these differences, the HbA1c level of ≥5.7% and fasting PG level of ≥94 mg/d in the Swedish study [20], and the HbA1c level of ≥5.6% and the 2-h PG level of ≥183 mg/dl in our study were not comparable to the marked hyperglycemia that is seen in patients with pregestational diabetes. It is therefore important to consider that those modestly elevated HbA1c and glucose levels (either the fasting or the post-glucose load) during pregnancy are associated with the development of diabetes within 5 years postpartum. Several studies addressed HbA1c as a parameter for predicting postpartum diabetes in women with GDM among different races and ethnicities with different diagnostic criteria for GDM and follow-up duration [24–27]. Those studies found significant independent predictive cut-off values for the development of diabetes between 5.4% and 5.7%. Again, a modestly elevated level HbA1c seems to be a significant predictor regardless of race and ethnicity.

In our previous study, we reported that both a lower insulingenic index, which exhibits decreased early-phase insulin secretion and insulin therapy during pregnancy are independent predictors for abnormal glucose tolerance, including both prediabetes and diabetes, in the early postpartum period [5].

Because we only measured insulin in half of the subjects in the current study, we were not able to address insulin dynamics during pregnancy. Kwak et al. [28] reported the difference in the characteristics between diabetic women with a history of GDM who were diagnosed in the early and late postpartum period. Interestingly, they suggested that women with the early development of diabetes had more pronounced defects in their beta-cell function, which might be explained by differences in their genetic predisposition [28].

The major strength of this study was the relatively high follow-up rate of up to 86%, which was very similar regardless of the diagnostic criteria used. As already mentioned, the previously reported postpartum follow-up rates were less than 50%. In our study, 75% of the subjects underwent at least two follow-up OGTTs and more than 50% of them were followed up beyond 12 months after their pregnancy (Table 2).

The present study is associated with several limitations. We did not address any postpartum factors, including postpartum weight change and breastfeeding. An increase in weight during the postpartum period is known to be a significant risk factor for the development of diabetes [2, 29, 30]. Although we controlled for the baseline obesity and weight gain in pregnancy in the multivariate analysis, we did not investigate the effect of the postpartum weight changes on the development of diabetes during the follow-up period. Breastfeeding is also expected to be a predictor of the postpartum development of diabetes in the general population [31] and in women with a history of GDM [32–34]. However, we did not investigate this factor because we could not obtain sufficient data on the subjects’ breastfeeding practices due to the retrospective approach of our study. Because of the small sample size in this study, we were unable to conclude that other variables, including prepregnancy obesity and insulin therapy during pregnancy as well as fasting PG during the diagnostic OGTT for GDM, were not significant predictors for the development of postpartum diabetes. Because of the small sample size, we were unable to perform analyses limited to women were diagnosed under the IADPSG criteria. Although our results suggest that the IADPSG criteria are efficient at identifying women with GDM at risk of developing postpartum diabetes, further prospective cohort studies with a larger sample size are necessary to draw any definitive conclusions on this issue.

## Conclusions

In conclusion, despite the diagnostic criteria, in women with a history of GDM, the elevation of the HbA1c and 2-h PG levels during pregnancy, at the time of the diagnostic OGTT, was independently associated with the development of diabetes within 5 years postpartum. Thus, to make an early diagnosis of postpartum diabetes, it is important to carefully pay attention to pregnant women with HbA1c and 2-h PG levels that are higher than the above-mentioned cutoff points of 5.6% and 183 mg/dl, respectively, despite the use of insulin.

## Abbreviations

AUC: area under the curve
BMI: body mass index
CC: Carpenters-Coustan
CI: confidence interval
GA: gestational age
GDM: gestational diabetes
HbA1c: hemoglobin A1c
IADPSG: International Society of Diabetes and Pregnancy Study Group
JSOG: Japan Society of Obstetrics and Gynecology
OGTT: oral glucose tolerance test
PG: plasma glucose
ROC: receiver operating curve
RR: relative risk
SMBG: self-monitored blood glucose
WHO: World Health Organization
